# Thrombospondin1 Deficiency Attenuates Obesity-Associated Microvascular Complications in ApoE-/- Mice

**DOI:** 10.1371/journal.pone.0121403

**Published:** 2015-03-24

**Authors:** Hasiyeti Maimaitiyiming, Kate Clemons, Qi Zhou, Heather Norman, Shuxia Wang

**Affiliations:** 1 Department of Pharmacology and Nutritional Sciences, University of Kentucky, Lexington, Kentucky, United States of America; 2 Lexington Veterans Affairs Medical Center, Lexington, Kentucky, United States of America; The Ohio State University, UNITED STATES

## Abstract

Obesity is associated with insulin resistance and the increased development of vascular complications. Previously, we have demonstrated that thrombospondin1 (TSP1) regulates macrophage function and contributes to obesity associated inflammation and insulin resistance. However, the role of TSP1 in the development of obesity associated vascular complications is not clear. Therefore, in the current study, we investigated whether TSP1 deficiency protects mice from obesity associated micro as well as macro-vascular complications in ApoE-/- mice. In this study, male ApoE-/- mice and ApoE-/-TSP1-/- mice were fed with a low-fat (LF) or a high-fat (HF) diet for 16 weeks. We found that body weight and fat mass increased similarly between the ApoE-/-TSP1-/- mice and ApoE-/- mice under HF feeding conditions. However, as compared to obese ApoE-/- mice, obese ApoE-/-TSP1-/- mice had improved glucose tolerance, increased insulin sensitivity, and reduced systemic inflammation. Aortic atherosclerotic lesion formation was similar in these two groups of mice. In contrast, albuminuria was attenuated and kidney fibrosis was reduced in obese ApoE-/-TSP1-/- mice compared to obese ApoE-/- mice. The improved kidney function in obese ApoE-/-TSP1-/- mice was associated with decreased renal lipid accumulation. Together, these data suggest that TSP1 deficiency did not affect the development of obesity associated macro-vascular complication, but attenuated obesity associated micro-vascular complications.

## Introduction

Thrombospondin1 (TSP1) is a major component of platelet alpha granules and also expressed by a verity of cell types [1–6]. TSP1 exists as both a component of the extracellular matrix and as a soluble molecule found in various body fluids and in the cell culture conditioned medium. TSP1 was up-regulated in developing adipose tissue of mice with diet or genetically induced obesity [7]. TSP1 was also up-regulated in obese insulin resistant humans and associated with adipose inflammation and insulin resistance [8]. Moreover, studies from our lab and others demonstrated that TSP1 is an important mediator of obesity associated insulin resistance [9–11]. In addition to insulin resistance, obesity is associated with the increased incidence of cardiovascular complications including atherosclerosis (macrovascular complication) and kidney disease (microvascular complication). Previous studies from our lab suggest that TSP1 mediates obesity induced kidney dysfunction [12]. However, whether TSP1 plays a role in obesity induced macro-vascular complications such as aortic atherosclerotic lesion formation is unknown.

In the current study, we utilized TSP1 and ApoE double knock out mice to investigate the role of TSP1 in obesity associated vascular complications in a high fat diet induced obesity mouse model. Using this proatherogenic and hyperlipidemic mouse model, we demonstrated that TSP1 deletion had no effect on obesity development. However, obese ApoE-/-TSP1-/- mice had reduced systemic inflammation, improved insulin sensitivity, attenuated kidney fibrosis and improved renal function. These results are in agreement with our previous studies [9,12] and further confirm the protective effect of TSP1 deficiency on obesity associated micro-vascular complications. However, TSP1 deficiency had no effect on obesity induced aortic atherosclerotic lesion formation, suggesting that TSP1 may not be a major player in obesity related macro-vascular complications.

## Materials and Methods

### Ethics Statement

All experiments involving mice conformed to the National Institutes of Health Guide for the Care and Use of Laboratory Animals and were approved by the University of Kentucky Institutional Animal Care and Use Committee.

### Animal model

TSP1-/- mice (on C57BL6/J background, purchased from Jackson Laboratory) were crossbred with ApoE-/- mice (C57BL6 /J background, purchased from Jackson Laboratory) to generate ApoE-/-TSP1-/- mice and ApoE-/- mice. Three month old male ApoE-/- and ApoE-/-TSP1-/- mice were used in the study. These mice were housed in a temperature controlled room with a 12 hour light/dark cycle. Mice were fed a low fat (LF) (10% kcal as fat; D12450B; research Diets, Inc, NJ) or a high fat (HF) diet (60% kcal as fat; D12492, research Diet, Inc, NJ) for 16 weeks. These diets contain no added cholesterol. The total cholesterol content is about 0.002 and 0.003% by weight in LF and HF diet, respectively. Each group contained 10–12 mice.

### Glucose tolerance and Insulin tolerance tests

After 15 weeks of LF and HF-feeding, glucose tolerance was analyzed in animals after 6 h fasting. Following an intraperitoneal injection of glucose (1g/kg body weight), blood glucose concentrations were measured using a Glucometer at 0, 15, 30, 60, and 120 minutes after injection. For insulin tolerance test, insulin (0.5 unit/kg body weight) (Novolin R, Novo Nordisk InC.) was injected into mice intraperitonealy. Similarly, blood glucose levels were measured at 0, 15, 30, 60, and 120 minutes after injection to assess insulin’s effect.

### Assessments of body composition

EchoMRI (Echo Medical System) was used to evaluate body fat and lean content in mice after 16 weeks of LF or HF feeding as pervious described [9].

### Metabolic measurements and lipoprotein characterization

At the end of the study, mice were sacrificed. The blood was collected and plasma insulin, TNF-α, PAI-1, and resistin concentrations were measured using a mouse adipokine assay kit (Millipore). Plasma total cholesterol levels were measured by enzymatic colorimetric assay using a kit from Wako Chemicals. In addition, lipoprotein cholesterol distributions were evaluated in individual serum samples after fractionation by fast protein liquid chromatography (FPLC) gel filtration on a single Superose 6 column as described previously [13]. In brief, a Bio-Rad Biologic DuoFlow System with BioFrac Fraction Collector and Superose 6 10/300GL Column (GE Healthcare) was used. The elusion buffer contained 0.15 M NaCl and 1 mM EDTA. After loading of 50 μL plasma into the system, the flow rate should not exceed 0.5 ml/min. The fractionation was started at 10 ml and ended at 26 ml with each fraction size of 0.5ml. Total of 32 fractions were collected. Cholesterol concentrations from fractions 1–32 were further measured by using Cholesterol E Enzymatic Kit (WAKO).

### Quantification of atherosclerotic lesions

Mouse aortas were removed and fixed in freshly prepared 4% paraformaldehyde in PBS overnight at room temperature. After fixation, adventitial tissue was removed. Aortas were cut and the intimal surface was exposed, pinned, and photographed for en face measurements of atherosclerosis as described previously [14] by the help from Dr. Alan Daugherty’s group at University of Kentucky.

### Renal Function, Histology, Immunoblotting, and Immunohistochemical Staining

#### Renal function measurement

Twenty-four-hour mouse urine was collected before death. Urine albumin (Exocell) and urinary creatinine (Exocell) were measured according to the manufacturer's instructions. Urinary albumin-to-creatinine ratio (ACR) was calculated as: ACR = urine albumin/urine creatinine (μg/mg).

#### Renal histology

Kidneys were harvested, fixed in 10% neutral formalin, embedded in paraffin, and sectioned into 4-μm sections. After deparaffinization, tissue sections were rehydrated and stained by periodic acid-Schiff (PAS) reagent (Sigma). Based on PAS staining, pathological changes of glomeruli were examined under light microscope. Analysis of glomerular area was blindly performed using computer imaging software.

#### Immunoblotting

Kidney cortex was homogenized and equal amount of total protein was subjected to SDS-PAGE gel under reducing conditions and transferred onto nitrocellular membrane. After blocking, the membrane was incubated with anti-phospho-Smad3 or Smad3 antibodies (Cell Signaling) and then incubated with horseradish peroxidase-conjugated secondary antibody. The reaction was visualized using an enhanced chemiluminescence system (Pierce). Immunoblots were analyzed by scanning densitometry and quantified by Quantity One Gel Analysis software (Bio-Rad).

#### Renal immunohistochemical staining

Kidney tissue sections were deparaffinized in xylene, and were rehydrated in graded mixtures of ethanol/water. After blocking, slides were incubated with anti-Collagen IV (Research Diagnostics) for 1 h at room temperature. A negative control was included by substituting control IgG for the primary antibody. After washing, biotinylated secondary antibody was applied for 30 min and then an avidin-biotin-peroxidase complex was applied to the slides for additional 30 min. Vectastain ABC system (Vector Lab) was used for color development with DAB. Semiquantitative analyses of Collagen IV were blindly performed for the percentage of positive staining cells using computer imaging software.

### Renal Oil Red O Staining, Lipid Extraction, and Measurement

To determine renal accumulation of neutral fats, frozen kidney sections were prepared and stained with filtered Oil Red O (0.3% wt/vol) solution for 30 min at room temperature and then counterstained with hematoxylin. The stained kidney sections were imaged with a microscope. In addition, total lipids were extracted from kidneys as described previously[15] and measured using a kit from Wako Chemicals.

### Statistical analysis

Data are the mean ± SE. Differences between groups were determined by ANOVA followed by Turkey’s post hoc tests or Student’s t-test as appropriate. The significance level was p<0.05.

## Results

### TSP1 deficiency on ApoE-/- background does not affect the development of diet induced obesity

Studies have demonstrated that TSP1 is an important mediator of obesity associated insulin resistance [9–11] and also contributes to obesity induced kidney dysfunction [12]. However, whether TSP1 plays a role in obesity induced macro-vascular complications such as aortic atherosclerotic lesion formation is unknown. In the current study, we utilized a proatherogenic and hyperlipidemic mouse model (ApoE-/- mice) to investigate the role of TSP1 in obesity associated vascular complications.

**Fig 1:**
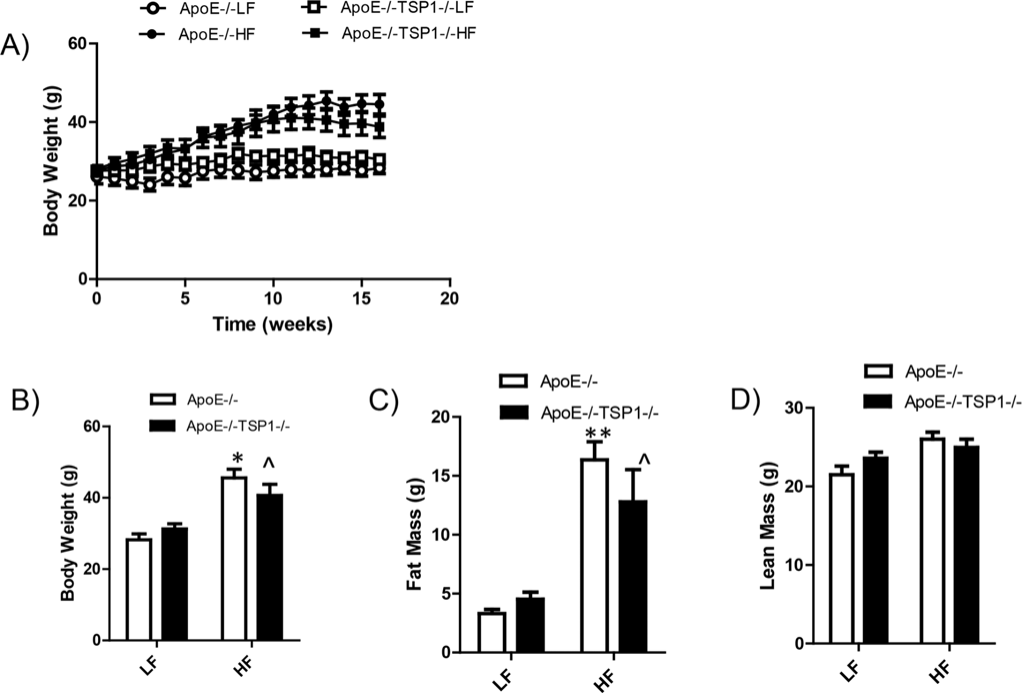
TSP1 deficiency had no effect on diet induced obesity in ApoE-/-mice. Male ApoE-/- mice and ApoE-/-TSP1-/- mice were fed LF or HF diet for 16 weeks. **A**): Graphs showing the increase of body weight over time on diets. **B**): body weight of mice at the end of the study. **C**) **and D**): Fat and lean mass of different groups of animals. Data are represented as mean ± SE (n = 10 mice/group). * p<0.05 or ** P<0.01vs. LF ApoE-/-; ^P<0.05 vs. LF TSP1-/-ApoE-/-

Previously, we showed that TSP1 deficiency on C57BL6 background did not affect diet induced obesity [9]. We determined whether this is also true on ApoE-/- background. After 16 weeks of high fat diet (HF) feeding, both ApoE-/- and ApoE-/-TSP1-/- mice showed almost comparable body weight and fat mass (Fig. 1), supporting the previous finding that TSP1 deficiency does not affect the development of obesity.

### HF-fed ApoE-/-TSP1-/- mice exhibit improved glucose tolerance and increased insulin sensitivity as compared to ApoE-/- mice, which is associated with reduced systemic inflammation

**Fig 2:**
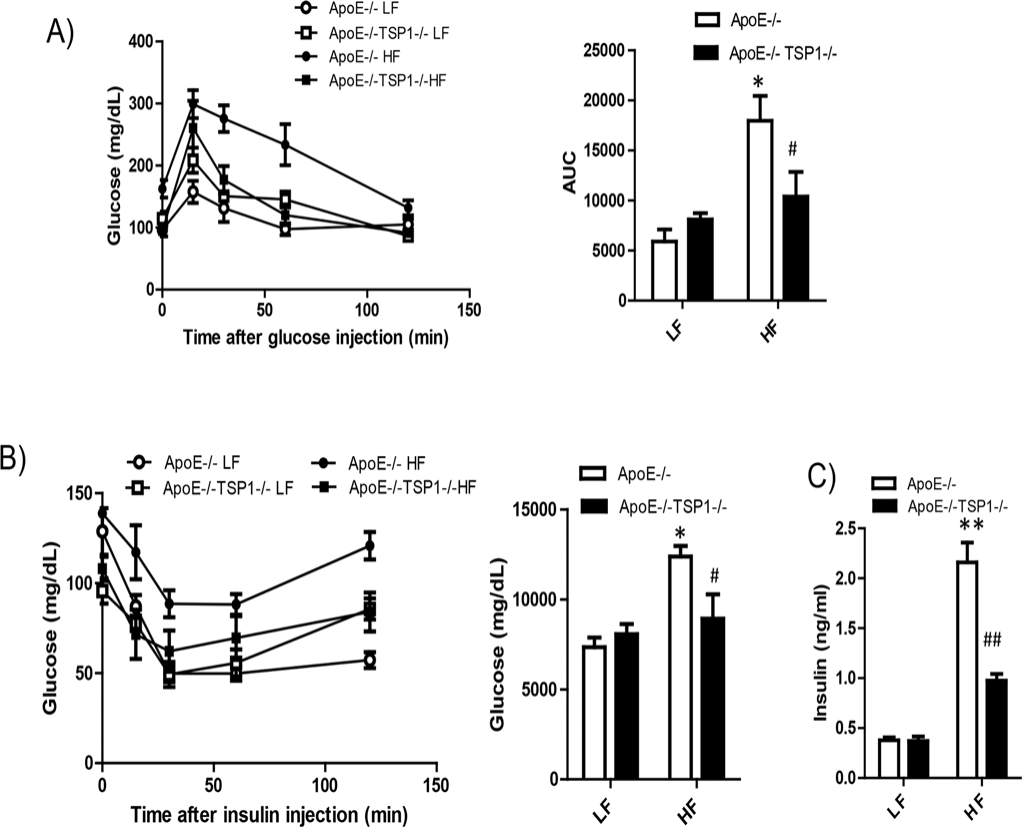
Obese ApoE-/-TSP1-/- mice had improved glucose tolerance and insulin sensitivity. Male ApoE-/-TSP1-/- mice and ApoE-/-mice were fed LF or HF diet for 16 weeks. Intraperitoneal glucose tolerance (A) and insulin tolerance test (B) were measured. Changes in blood glucose levels were monitored over time. C) Plasma insulin levels were measured by ELISA. Data are represented as mean ± SE (n = 10 mice/group). *P<0.05 and ** P<0.01 vs. LF ApoE-/-. # P<0.05 and ## P<0.01 vs. HF ApoE-/-. AUC: area under the curve.

**Fig 3:**
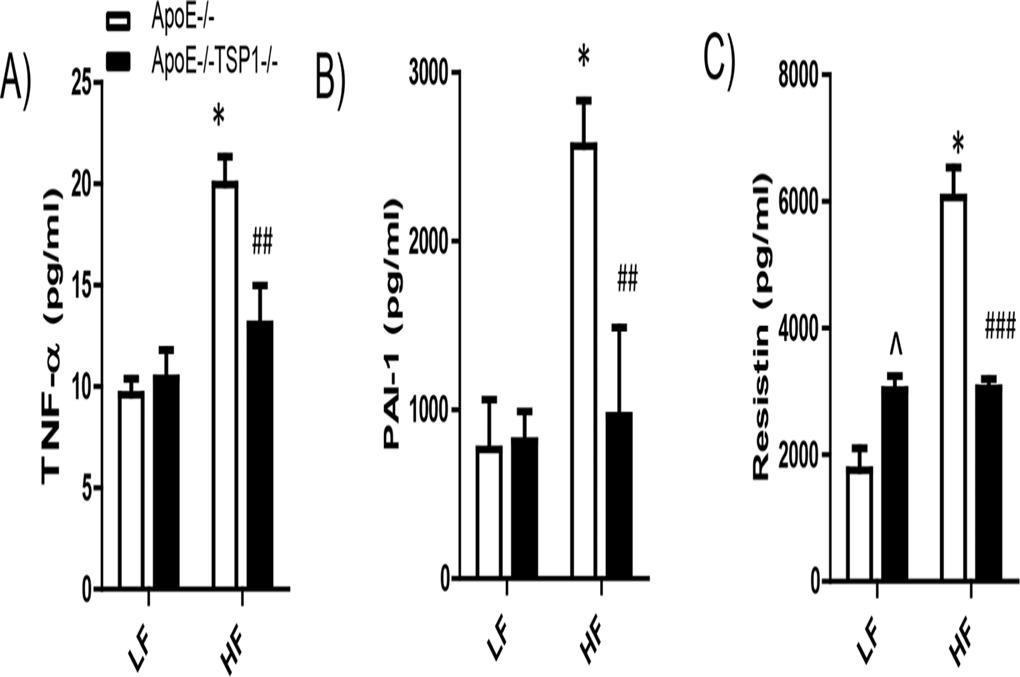
Obesity induced systemic inflammation was reduced in ApoE-/-TSP1-/- mice. Plasma TNF-α, PAI-1 and resistin levels were measured as described in Material and Methods section. Data are represented as mean ± SE (n = 6 mice/group). ^ P<0.05 vs. LF ApoE-/-; *P<0.05 vs. LF ApoE-/-; ## P<0.01 or ### P<0.001 vs. HF ApoE-/-.

We determined whether TSP1 deficiency protected mice from obesity associated insulin resistance in ApoE-/- mice. Fasting insulin levels were measured. Glucose tolerance test (GTT) and insulin tolerance test (ITT) were performed in LF and HF fed mice. GTT and ITT tests demonstrated that HF-fed ApoE-/-TSP1-/- mice had improved glucose tolerance and insulin sensitivity (Fig. 2). Although HF feeding increased the insulin levels in both genotypes, the insulin was increased to a significantly lower extent in ApoE-/-TSP1-/-mice as compared to ApoE-/- mice (Fig. 2). In addition, obesity significantly increased plasma TNF-α, PAI-1 and resistin levels in ApoE-/- mice but not in ApoE-/-TSP1-/- mice (Fig. 3). Together, these data suggested that TSP1 deficiency reduced obesity associated inflammation and improved glucose homeostasis.

### ApoE-/-TSP1-/- mice have similar levels of aortic atherosclerotic lesion formation compared to ApoE-/- mice

**Fig 4:**
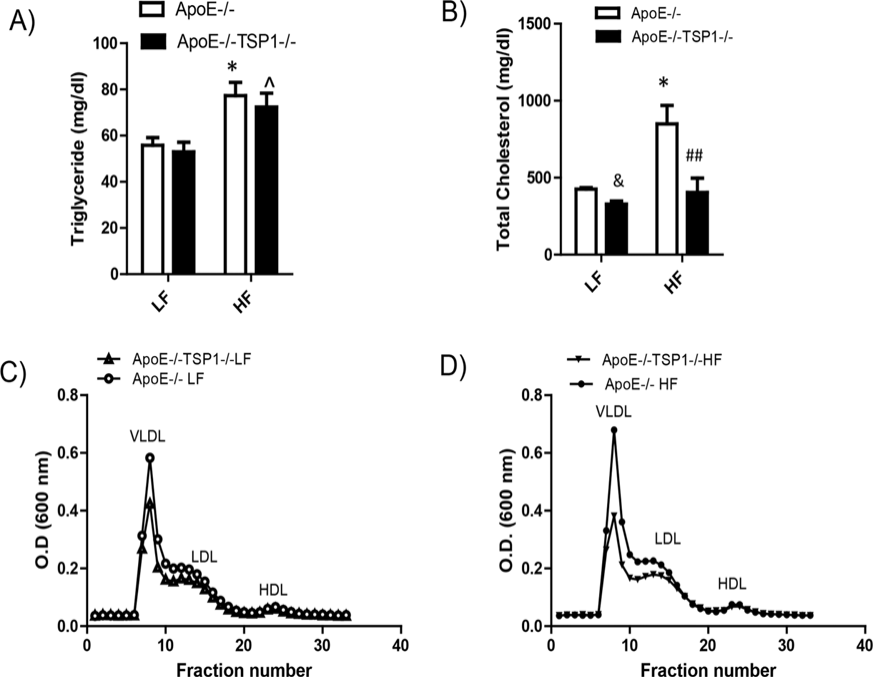
Effect of TSP1 deficiency on plasma triglyceride levels, total cholesterol levels, and cholesterol lipoprotein distribution under either LF or HF feeding conditions. Male ApoE-/-TSP1-/- mice and ApoE-/-mice were fed LF or HF diet for 16 weeks. A). Plasma triglyceride levels were measured. B). Plasma total cholesterol levels were measured. C) and D) cholesterol lipoprotein distribution was analyzed by FPLC. Data are represented as mean ± SE (n = 6 mice/group). & P<0.05 vs, LF ApoE-/-; *P<0.05 vs. LF ApoE-/-. ## P<0.01 vs. HF ApoE-/-. ^ p<0.05 vs. LF ApoE-/-TSP1-/-

**Fig 5:**
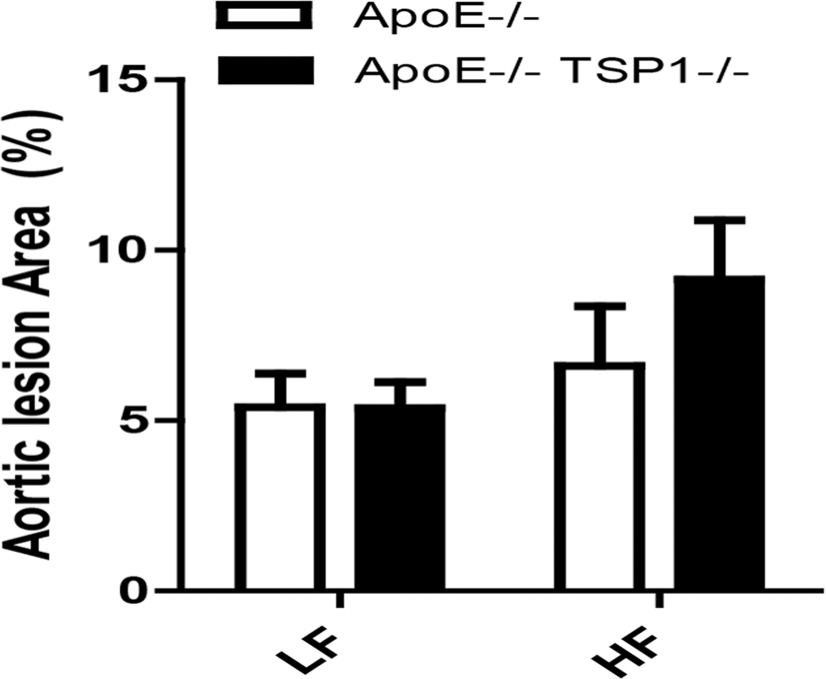
Effect of TSP1 deficiency on aortic lesion formation. Male ApoE-/-TSP1-/- mice and ApoE-/-mice were fed LF or HF diet for 16 weeks. Aortic lesion formation was analyzed by en face method. Data are represented as mean ± SE (n = 6–10 mice/group).

We determined the effect of TSP1 deficiency on obesity associated atherosclerosis development. First, we examined the levels of plasma triglyceride (TG), total plasma cholesterol and cholesterol lipoprotein distribution in four groups of mice. We found that under LF feeding conditions, plasma TG levels were comparable between ApoE-/- and ApoE-/-TSP1-/- mice. HF diet feeding similarly increased plasma TG levels in ApoE-/- mice and ApoE-/-TSP1-/- mice (Fig. 4). In addition, high fat feeding significantly increased plasma total cholesterol levels in ApoE-/- mice, but not in ApoE-/-TSP1-/- mice. Moreover, fast performance liquid chromatography (FPLC) analysis of cholesterol lipoprotein distribution showed that ApoE-/-TSP1-/- mice had reduced plasma VLDL and LDL levels (Fig. 4). Next, we determined the aortic atherosclerotic lesion formation in mice by utilization of en face method[14]. Unexpectedly, we found that high fat diet feeding did not increase the aortic atherosclerotic lesion formation in both ApoE-/- and ApoE-/-TSP1-/- mice. Moreover, aortic atherosclerotic lesion formation was similar between ApoE-/- mice and TSP1-/-ApoE-/- mice under either LF or HF feeding conditions (Fig. 5), suggesting that TSP1may not be a major player in the development of atherosclerosis under either normal or obese conditions.

### TSP1 Deficiency protects ApoE-/- mice from obesity-induced kidney dysfunction

**Fig 6:**
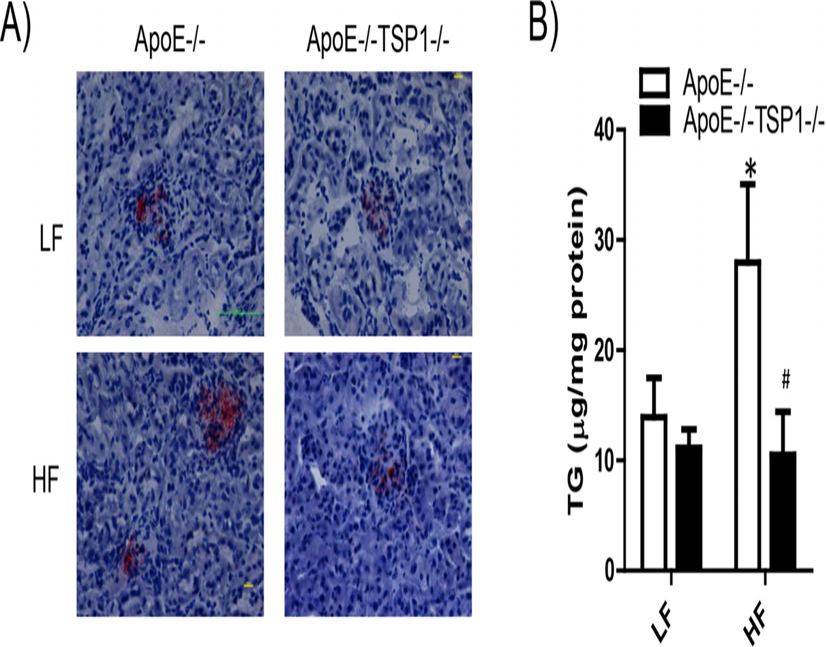
TSP1 deficiency reduced lipid accumulation in the kidney in ApoE-/- mice under HF feeding conditions. (A). Representative photograph of Oil red O staining of frozen kidney sections from 4 groups of mice. Lipid droplets were shown as red spots. (B). Triglyceride contents were measured in lipid extracts from kidney samples. Data are represented as mean ± SE (n = 6 mice/group). *P<0.05 vs. LF ApoE-/-. # P<0.01 vs. HF ApoE-/-.

**Fig 7:**
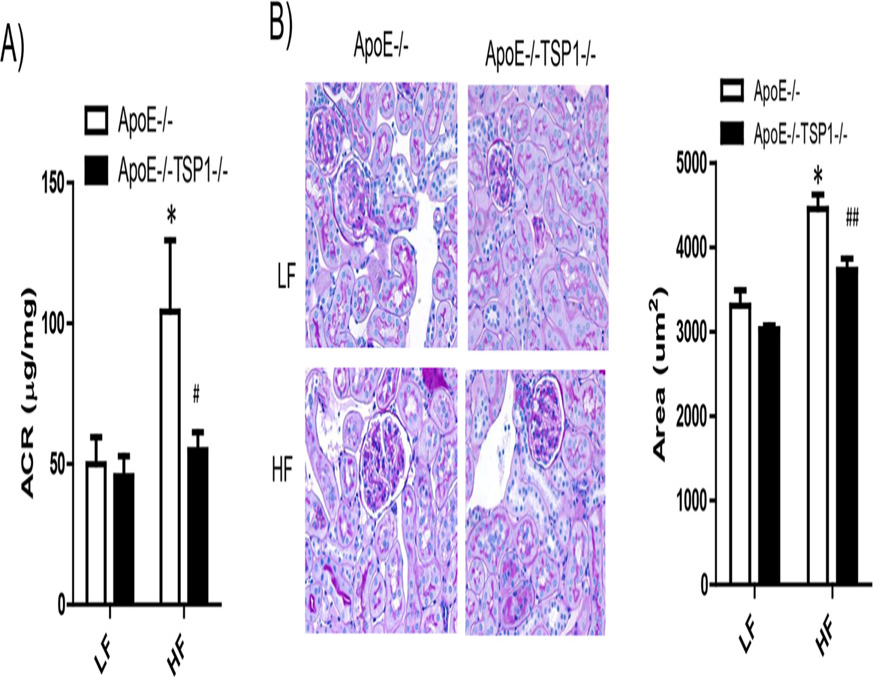
TSP1 deficiency attenuated obesity associated kidney dysfunction in ApoE-/- mice. A). Urinary albumin-to-creatinine ratio (ACR) was examined. B). Representative light micrographs of periodic acid-Schiff (PAS) stained kidney sections from 4 groups of mice. C) Glomerular area was analyzed by computer image analysis software. * P<0.05 vs. LF ApoE-/-. # P<0.05, ## P<0.01vs. HF ApoE-/-.

**Fig 8:**
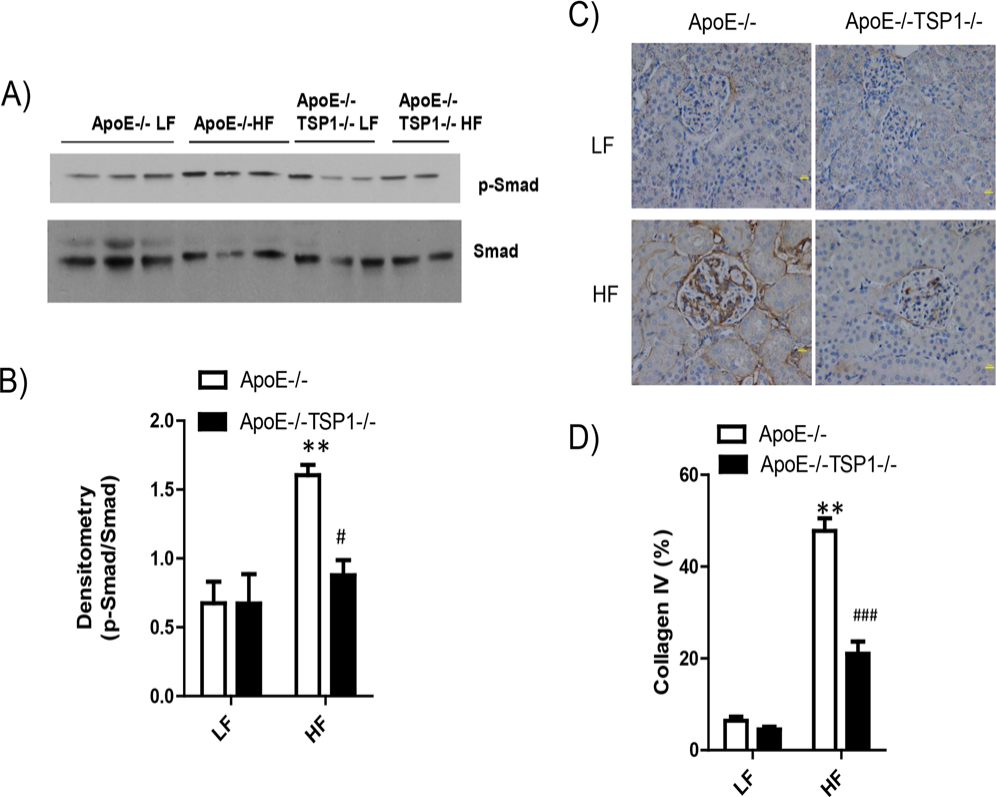
TSP1 deficiency attenuated obesity associated kidney fibrosis in ApoE-/- mice. A) and B). Immunoblotting of p-Smad and Smad levels in kidney cortex homogenates from four groups of mice. C) kidney collagen IV levels were detected using immunohistochemical staining, followed by semiquantitative analysis (D). Data are represented as mean ± SE (n = 6–10 mice/group). ** P<0.01 vs. LF ApoE-/-. # P<0.05, ### P<0.001vs. HF ApoE-/-.

We determined the effect of TSP1 deficiency on the development of obesity-induced kidney damage (a microvascular complication) in ApoE-/- mice. As shown in Fig. 6, kidney lipid accumulation was significantly increased in HF-fed ApoE-/- mice but not in HF-fed ApoE-/-TSP1-/- mice, determined by Oil Red O staining of kidney sections and renal triglyceride content. In addition, obese ApoE-/- mice wild-type mice developed albuminuria and renal hypertrophy (Fig. 7). Phospho-Smad levels (TGF-β downstream signaling) were increased in obese ApoE-/- mice, which was associated with increased renal fibrosis in these mice (Fig. 8). In contrast, HF diet feeding-induced phospho-Smad levels were reduced in obese ApoE-/-TSP1-/- mice and these mice had attenuated renal damages. Together, these data suggest that TSP1 deficiency protects mice from pro-atherogenic and hyperlipidemia induced kidney injury.

## Discussion

In the present study, we have determined the effect of TSP1 deficiency on the development of obesity and hyperlipidemia induced macro and micro-vascular complications in ApoE-/- mice.

The results demonstrate that TSP1 deficiency improves metabolic phenotype of HF-fed ApoE-/- mice with reduced obesity-associated systemic inflammation and improved insulin sensitivity. TSP1 loss is associated with the significant reduction in lipid accumulation in kidney and attenuated obesity associated kidney dysfunction. In contrast, the development of aortic atherosclerotic lesion formation is not altered by TSP1 deletion. Together, these data suggest that TSP1 is an important player in obesity and hyperlipidemia induced microvascular complications.

The ApoE null mouse is a well-established model for studying atherosclerosis (macrovascular disease) as well as hyperlipidemic renal injury (microvascular disease) [16]. Recently, high fat diet (60% fat) fed ApoE-/- mice has been demonstrated to be an animal model of obesity induced accelerated atherosclerosis [17]. Therefore, using this unique mouse model, we determined the role of TSP1 in pro-atherogenic and hyperlipidemia induced cardiovascular and renal complications. Previous studies from our lab and others using TSP1 deficient mice (on C57 BL6 background) suggest that TSP1 contributes to obesity associated inflammation and insulin resistance [9–11]. Consistently, in the current studies, by using TSP1 deficient mice on ApoE-/—background, our data confirmed and extended the previous findings [9]. We showed that TSP1 deficiency did not affect the development of diet induced obesity in ApoE-/- mice. Although ApoE-/-TSP1-/- mice developed similar level of obesity as ApoE-/- mice, ApoE-/-TSP1-/- mice had reduced systemic inflammation and improved glucose tolerance and insulin sensitivity. These data further support the role of TSP1 in obesity associated insulin resistance.

The current studies demonstrate that TSP1 deficiency improved the metabolic phenotype of diet induced obese ApoE-/- mice. However, whether this improved metabolic phenotype leads to attenuated cardiovascular complications in these mice is unknown. Therefore, in the current studies we evaluated the development of atherosclerosis in ApoE-/-mice. It has been shown from King et al that ApoE-/- mice developed increased atherosclerosis after 17 weeks of high fat diet feeding [17]. Unexpectedly, in our studies, we did not reveal increased atherosclerotic lesion formation in high fat diet fed ApoE-/- mice. The reason for causing this discrepancy is unknown. The factors including different batch of diet, duration of high fat diet feeding and animal ages may contribute to this different finding. In our study, we fed 12 week old male ApoE-/- mice with LF or HF diet for 16 weeks; whereas King et al fed 8 week old male ApoE-/- mice with LF or HF diet for 17 weeks. In addition, other unknown factors may also play a role.

Multiple factors contribute to the development of atherosclerosis. Levels of plasma cholesterol and cholesterol lipoprotein distribution are major risk factors for atherosclerosis formation. Interestingly, we found that high fat diet feeding significantly elevated total plasma cholesterol levels in ApoE-/- mice. However, in ApoE-/-TSP1-/- mice, high fat diet had no effect on plasma total cholesterol levels. Moreover, under either low fat or high fat feeding conditions, ApoE-/-TSP1-/- mice had reduced levels of pro-atherogenic lipoproteins: VLDL and LDL. HDL levels were similar between ApoE-/- and ApoE-/-TSP1-/- mice. According to our knowledge, this is the first report showing the effect of TSP1 on alteration of cholesterol lipoprotein distribution. The mechanism is unknown at this time and will be investigated in the future. In addition to this novel effect of TSP1 on lipid profile regulation, a variety of other activities have been ascribed to TSP1 from in vitro studies such as inducing endothelial cell dysfunction[18], stimulating smooth muscle cell proliferation and migration[19,20] and activating latent TGF-β activation [21–23]. All of these TSP1-mediated activities suggest that TSP1 is pro-atherogenic. However, in the current study, TSP1 deficiency did not reduce/prevent atherosclerotic lesion formation. ApoE-/-TSP1-/- mice and ApoE-/- mice showed comparable aortic lesion area under either low fat or high fat diet feeding conditions. This result is in agreement with a previous report from Moura et al showing that TSP1 deficiency only had minimal effect on the atherosclerotic lesion formation in ApoE-/- mice under normal chow diet feeding conditions [24]. Moreover, they found that TSP1 deficiency modulated the intra-plaque composition. ApoE-/-TSP1-/- plaque contained less contractile smooth muscle cells, more collagen and more macrophage numbers compared to plaque from ApoE-/- mice. This phenomenon is independent of TSP1-mediated TGF-β activation, since TGF-β signaling (Smad phosphorylation) in vascular extracts was comparable in both genotypes. Their studies further suggested a role for TSP1 in the plaque maturation during the lesion progression [24]. Taken together, studied from ours and others suggest that the in vivo effect of TSP1 on atherosclerotic lesion development (plaque formation or maturation) is complex [25] and warrants further investigation.

Accumulating evidence suggests the role of TSP1 in the development of diabetic nephropathy, a microvascular disease [26–28]. TSP1 is a major regulator for the well-known fibrogenic growth factor-TGF-β [21,22,29–31]. Most cells secrete TGF-β as an inactive precursor and must be converted to an active form that can bind to its receptors and elicit a cell response. TSP1 is one of the major physiological regulators of latent TGF-β activation [21–23]. TSP1-mediated latent TGF-β activation has been found in vitro as well as in vivo in experimental diabetic nephropathy [27,32–36]. Our recent studies showed that TSP1 deficiency ameliorates obesity-associated kidney dysfunction [12]. However, whether TSP1 deficiency protects kidney function in pro-atherogenic hyperlipidemic mouse model has not been explored. Many factors such as hyperlipidemia, hypertension, chronic inflammation, or decreased adiponectin levels can contribute to obesity associated kidney disease [37–39]. In the current study, we found that under LF feeding conditions, plasma triglyceride (TG) levels were comparable between ApoE-/- and ApoE-/-TSP1-/- mice. HF diet feeding similarly increased plasma TG levels in ApoE-/- mice and ApoE-/-TSP1-/- mice. Although TSP1 deficiency did not affect circulating TG levels in either LF or HF fed mice, TG levels in the kidney tissue were significantly reduced in HF-fed ApoE-/-TSP1-/- mice compared to HF-fed ApoE-/- mice. However, kidney TG levels were comparable between LF-fed ApoE-/-TSP1-/- mice and LF-fed ApoE-/- mice. This suggests that TSP1 may specifically regulate obesity/ HF feeding associated lipid dys-metabolism in the kidney. Lipid accumulation in kidney has been described in both obese animal models and human subjects [40,41]. Several key genes in lipid metabolism such as sterol regulatory element binding protein (SREBP-1), LDL receptor, or fatty acid binding protein 3 were significantly increased in the kidney from obese patients [42]. Therefore, we speculate that TSP1 may regulate the expression of some of these key genes in the kidney, which affects lipid synthesis, metabolism, or export, contributing to obesity associated renal injury. The contribution of renal lipid accumulation to renal dysfunction has been well established [41]. In the current study, with reduced renal lipid accumulation, high fat diet—fed ApoE-/-TSP1-/- mice had reduced albuminuria and glomerular hypertrophy. Moreover, these mice had reduced active TGF-β signaling in the kidney and developed less kidney fibrosis (reduced collagen IV production). Together, these data suggest that TSP1 plays an important role in the development of obesity associated microvascular complications.

In summary, by utilization of the pro-atherogenic hyperlipidemic mouse model in the current study, our data support the important role for TSP1 in obesity-induced inflammation and insulin resistance. Moreover, our data suggest that TSP1 is an important player in obesity and hyperlipidemia induced microvascular complications.

## Supporting Information

S1 Fig Photograph of Oil red O staining of frozen kidney sections from ApoE-/-LF and ApoE-/-HF feeding mice (n = 3 mice/group). (TIF)

S2 Fig Photograph of Oil red O staining of frozen kidney sections from ApoE-/-TSP1-/-LF and ApoE-/-TSP1-/-HF feeding mice (n = 3 mice/group). (TIF)
