# Supplementary figures and images for: Thrombospondin1 Deficiency Attenuates Obesity-Associated Microvascular Complications in ApoE-/- Mice
Source: PLoS One. 2015 Mar 24;10(3):e0121403. doi: 10.1371/journal.pone.0121403 (PMC4372557; doi:10.1371/journal.pone.0121403)

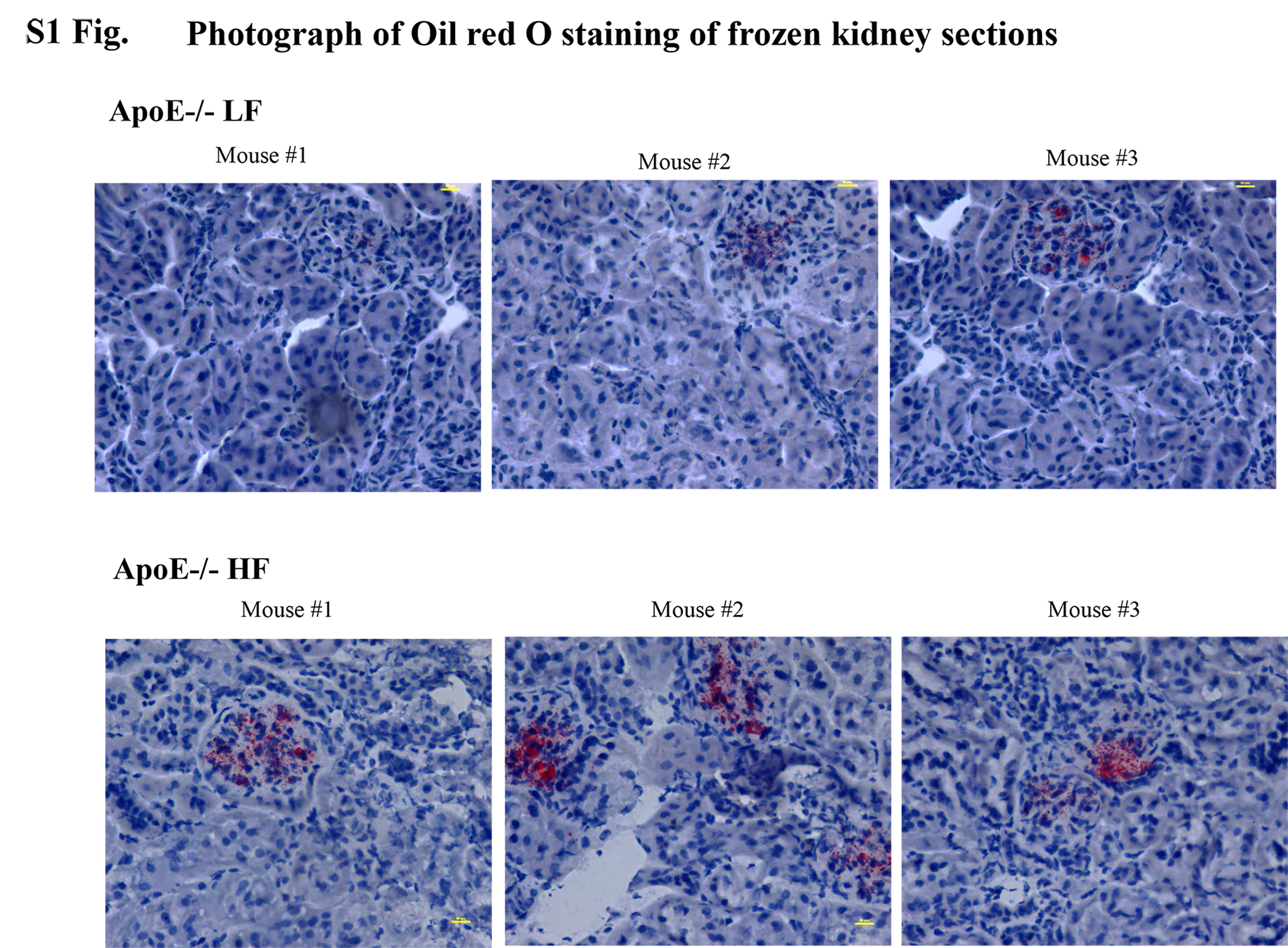

Supplement: S1 Fig — (TIF) [file pone.0121403.s001.tif]

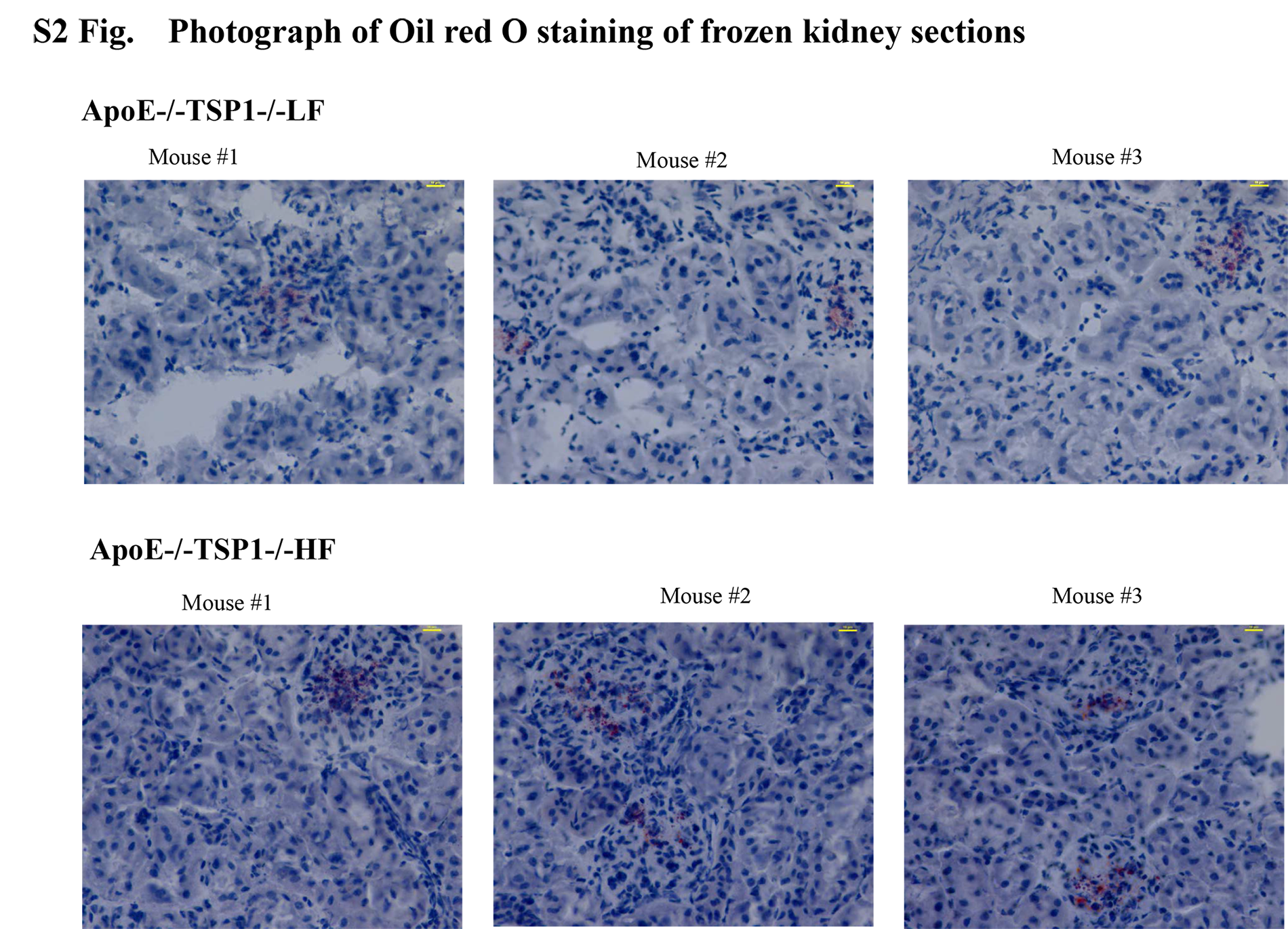

Supplement: S2 Fig — (TIF) [file pone.0121403.s002.tif]
